# Future-Proofing Startups: Stress Management Principles Based on Adaptive Calibration Model and Active Inference Theory

**DOI:** 10.3390/e23091155

**Published:** 2021-09-02

**Authors:** Stephen Fox

**Affiliations:** VTT Technical Research Centre of Finland, FI-02150 Espoo, Finland; stephen.fox@vtt.fi; Tel.: +358-40-747-8801

**Keywords:** active inference theory (AIT), adaptive calibration model (ACM), double-loop learning, conservation of resources, free energy principle, physics of life, startups, stress, triple-loop learning

## Abstract

In this paper, the Adaptive Calibration Model (ACM) and Active Inference Theory (AIT) are related to future-proofing startups. ACM encompasses the allocation of energy by the stress response system to alternative options for action, depending upon individuals’ life histories and changing external contexts. More broadly, within AIT, it is posited that humans survive by taking action to align their internal generative models with sensory inputs from external states. The first contribution of the paper is to address the need for future-proofing methods for startups by providing eight stress management principles based on ACM and AIT. Future-proofing methods are needed because, typically, nine out of ten startups do not survive. A second contribution is to relate ACM and AIT to startup life cycle stages. The third contribution is to provide practical examples that show the broader relevance ACM and AIT to organizational practice. These contributions go beyond previous literature concerned with entrepreneurial stress and organizational stress. In particular, rather than focusing on particular stressors, this paper is focused on the recalibrating/updating of startups’ stress responsivity patterns in relation to changes in the internal state of the startup and/or changes in the external state. Overall, the paper makes a contribution to relating physics of life constructs concerned with energy, action and ecological fitness to human organizations.

## 1. Introduction

Startups are founded by entrepreneurs with the aim of developing scalable enterprises. However, despite startup founders aiming for growth, the failure rate of startups is approximately 90 percent. Thus, rather than grow, typically only one out of ten startups survive. Moreover, predicting which startup will be successful is so difficult that it can be more effective to allocate startup funding randomly rather than on the basis of analyzing startups’ plans [1,2,3,4]. Accordingly, new perspectives are needed to better enable startups’ survival and growth: especially amidst the challenges introduced by widespread climate-related environmental changes [5,6,7,8,9,10]. Here, future-proofing is relevant. This involves anticipating future challenges and applying methods to minimizes their effects [11,12,13,14,15].

A long established method for improving the performance of organizations is the definition of principles that can guide operations successfully in a wide variety of settings [16,17]. In 2021, it is argued that, in order to avoid exacerbating climate change and to develop the resilience of human enterprises to climate change, nature-based methods are preferred [18,19]. Hitherto, however, underlying principles that guide the organization of nature have not been considered as a basis for providing principles for future-proofing startups. This is despite the nature-based term, ecosystem, being widely used in connection with startups [20,21]. By contrast, in this paper, the Adaptive Calibration Model (ACM) [22] and Active Inference Theory (AIT) [23] are related to the future-proofing of startups.

ACM addresses the allocation of finite time and energy by the stress response system to alternative options for actions, depending upon individuals’ life histories and changing external contexts [22]. ACM is relevant to organizational life cycles from an entrepreneur with a startup idea through to the few startups that grow to become large organizations. For example, the need to manage stress is recognized in literature concerned with entrepreneurs [24,25] and large organizations [26,27]. Active Inference Theory (AIT) is a physics of life process theory [23], which is a corollary of the Free Energy Principle (FEP) [28]. Within AIT, living things, including humans, implement internal generative models in order to survive. This involves humans surviving by taking action to align their internal generative models with sensory inputs from external states. In accordance with FEP, this is done to address the existential need for active systems to minimize the long-term average of unwanted surprise from external states: i.e., from the world [28]. Within stress studies incorporating AIT, stress arises from existential information entropy in trying to align the internal state with the external state: i.e., stress arises from uncertainty about survival [29,30]. Apropos, stress can lead to the collapse of higher goals in internal generative models [29], and stress can lead to the formulation of maladapted internal generative models with negative prior expectations that override positive sensory inputs [30].

As explained in the following sections, ACM and AIT can provide physics of life principles for addressing the potential of the stress response system to support or to undermine survival through action. ACM and AIT are related to future-proofing startups in the five remaining sections. In Section 2, ACM is related to life cycle phases of startups. In Section 3, ACM and AIT are related to business model development and marketing. In Section 4, ACM and AIT is related to startup practice in terms of the need for living things to maintain non-equilibrium steady states (NESS). In Section 5, stress management principles for the future-proofing of startups based on ACM and AIT are proposed. In Section 6, principal contributions are stated and directions for further research are proposed. ACM and AIT are applied together in this paper because under active inference, self-organizing systems must select between alternative courses of action based upon their expected potential to align sensory data predicted by the internal generative models with those data generated by external states. However, AIT does not define what are the sequences of stress management actions (i.e., policies) that the self-organizing system can select between. Hence, ACM complements AIT by offering a defined set of stress responsivity patterns that do offer stress management policies that a startup can select between.

Three contributions are intended from the paper. First, to make a contribution to addressing the need for future-proofing methods for startups. Second, to relate ACM and AIT to startup life cycles in order to provide principles for recalibrating/updating stress response patterns to changes in startups’ internal states and changes in the external state within which startups seek to survive. This is different to previous studies that have focused on particular stressors [24,25,26,27]: rather than on recalibration/updating of stress responsivity patterns. Third, to provide practical examples that show the broader relevance ACM and AIT to organizational practice. Previous research has explained the relevance of AIT to human organizations that offer high volume low variety goods and/or services: e.g., mass production organizations deploying quality management systems. However, previous research has highlighted the need to for further investigation of AIT’s relevance to human organizations [31]. Overall, the paper makes a contribution to relating physics of life constructs concerned with energy, action, and ecological fitness to practice in human organizations.

## 2. Adaptive Calibration Model (ACM): Startup Life Cycle

ACM addresses ways by which living things deploy stress response systems to allocate finite time and energy throughout their life cycle in order to survive [22]. Startups can have four lifecycle stages: business model development, transition, scaling, and exit. During business model development, organization is typically informal. During transition, the loosely structured informality of the initial stage changes to a more structured form that can facilitate rapid scaling. During the scaling phase, functional specialists take roles once covered by generalists, and formal procedures replace ad hoc decision making. At some point, an “exit” is undertaken through initial public offering of shares, private sale, merger, or acquisition [32]. In terms of ecological fitness, these four stages can be considered as two phases: arrival of the fittest and survival of the fittest [33]. In particular, the arrival of the species with the highest ecological fitness for an environmental niche is brought about by natural innovation processes. Subsequently, the survival of the fittest takes place through preservation of fittest through natural selection of useful adaptations. Ecological fitness is the potential of living things to survive in environmental niches through competition, cooperation, and/or construction that changes the environment [34].

At the outset of a startup when it is one person with an idea, the startup’s stress response system can be its founder’s stress response system, which will involve interactions between the person’s the sympathetic system, parasympathetic system, and hypothalamic-pituitary-adrenal axis (HPA). The sympathetic nervous system prepares the body for the “fight or flight” response during any potential danger. This is complemented by the parasympathetic nervous system that inhibits the body from overworking and restores the body to a calm and composed state [35]. Meanwhile, the HPA controls reactions to stress and regulates many body processes, including energy storage and expenditure. As the life cycle of a startup progresses, these functions need to be replaced by well-resourced formal operating procedures [27,36].

The allocation of time and energy during life cycle stages is crucial as both are limited. Hence, there are trade-offs between their allocation to different components of ecological fitness. For example, between investing in exploitation of existing information to promote current market offerings versus investing in exploration for new information to enable new market offerings. Within ACM, each trade-off is a decision node in allocation of resources, and each decision node influences the next decision node, which opens up some options while closing off other options, in a chain over the life course [37].

Within ACM, there is no one best life course. Rather there is adaptive developmental plasticity that involves interaction between internal and external variables. With regard to internal variables, the stress response system acts as an integrative mechanism, which mediates the development of alternative life strategies that are adaptive in different environmental conditions. This leads to conditional adaptive developmental variation through what is described in organizational studies as double-loop learning [38]. In particular, information encoded by the stress response system during development feeds back on the long-term calibration of the system itself. This double-loop learning results in adaptive patterns of stress responsivity, stress appraisals, and consequent individual differences in life history-related behavior. With regard to changing external variables, at different locations at different times there can be changing resource availability, environment characteristics, and uncertainty. The purpose of adaptive developmental plasticity is to maximize ecological fitness [22]. In terms of ACM, the move from one life cycle stage to the next can be switch points for the calibration of stress responsivity. For example, in the life cycle of startups when there are crises of leadership and bureaucracy during moving from informal to formal structure [32].

Through the stress response system, Adaptive Calibration Model (ACM) addresses the coordination of allostatic responses. Allostasis is the process of achieving internal stability through physiological or behavioral change—in contrast with homeostasis, which maintains internal stability by maintaining the organism’s internal state at a set point [22]. Also, ACM encompasses the encoding and filtering of information from the environment, thus mediating openness to environmental inputs. In particular, the stress response system continuously “samples” the environment, and its pattern of activation over the years provides a representation of key dimensions of the environment, which can then be used to orient the individual’s developing life history strategy. Different strategies may require different calibrations of the stress response system. In addition, ACM encompasses the regulation of traits and behaviors that can affect ecological fitness [22].

Within ACM, four prototypical patterns of stress responsivity are posited: sensitive (I), buffered (II), vigilant (III), and unemotional (IV). Sensitive pattern (I) is characterized by openness to the physical and social environment, which enable rapid adjustment to temporary perturbations in the environment through low risk cooperative life history strategies. Buffered pattern (II) is characterized by low-to-moderate stress responsivity in active engagement with the social environment involving long-term relationships. Buffered pattern is distributed widely across environmental conditions compared to sensitive pattern that is more likely in protective, low-stress developmental contexts. Vigilant pattern (III) develops in stressful contexts, where they enable people to cope effectively with dangers and threats in the physical and social environment. In the vigilant pattern, psychological resources are employed to monitor and cope with possible sources of threat and/or social competition, rather than to maximize learning and relaxed exploration as in the sensitive pattern (I). In the unemotional pattern (IV), there is generalized unresponsivity that inhibits social learning and sensitivity to social feedback, which can also increase risk-taking by blocking information about dangers and threats in the environments. Different stress responsivity patterns can lead to different appraisals of the same event: i.e., different stress appraisals of the same event [22].

**Figure 1:**
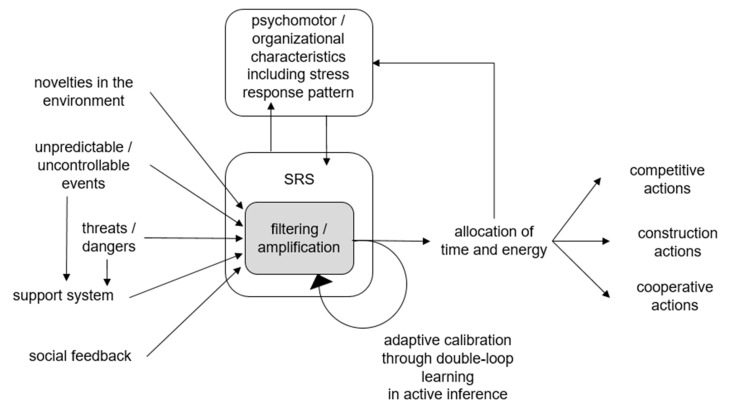
Adaptive Calibration Model for Startups.

A summary of ACM for startups is provided in Figure 1. Based on [22], this diagram shows that the stress response system (SRS) filters and/or amplifies unpredictable/uncontrollable events and threats/dangers in relation to support system that can offset them. At the same time, SRS filters and/or amplifies novelties in the environment and social feedback. Filtering and/or amplification regulate life history traits in terms of decisions about allocation of time and energy to competitive actions, cooperative actions, and/or construction actions. Initially, SRS is situated amidst psychomotor characteristics of founders. Subsequently, SRS is situated with organizational characteristics such as policy statements. Throughout, these include stress responsivity patterns (I, II, III, or IV), which affect and are affected by the SRS as it is calibrated through double-loop learning during active inference. In the following sections, adaptive calibration through active inference is related to startup lifecycle stages.

## 3. Business Model Development and Transition: Arrival of the Fittest

### 3.1. Business Model Development: Formulation of Internal Generative Model

The first phase of a startup can involve five activities [39]. First, formulating falsifiable hypotheses about a startup idea. Second, embedding these hypotheses into a designed business model. Third, developing a minimum viable product in order to test the business model. Fourth, customer development through identifying “earlyvangelist” customers from whom to receive feedback that can inform discovery and/or creation of many more customers. Fifth, running tests with multiple iterations to make decisions about whether or not to persevere with the startup idea. Together, these five activities span build-measure-learn-feedback that can lead to the startup having a well-defined business model encompassing its value proposition, market segments, cost structure, revenue streams, etc., [39]. Common across the five activities are opportunity creation, effectuation, and bricolage. In startup scholarship concerned with opportunity creation, opportunities for entrepreneurial profit are formed endogenously through action amidst uncertainty where outcomes are difficult to determine [40,41]. Similarly, within scholarship concerned with effectuation, opportunities are often endogenous to actors who focus on what can be done to move toward a yet-to-be-determined near-term future end point [42,43]. Additionally, within scholarship concerned with bricolage, actors are creative. In particular, they use whatever is at hand to create new solutions to problems as they arise [44,45]. The need for action is common across lean startup, opportunity creation, effectuation, and bricolage. The importance of action is further emphasized in scholarship that reports on action theory and action learning [46,47].

Similarly, action is at the core of AIT [48]. In particular, humans survive by taking action to reduce information gaps between their internal models and reality [30]. This involves actions being taken with the aim of aligning the sensory data predicted by the internal generative models with those data generated by external states. Better aligning includes formulating new internal generative models through insights gained via curiosity in exploring new external states [49]. For other livings things, such exploration may require being physically present in the new external state. By contrast, humans can undertake curiosity-driven exploration without being physically present in the new external state: for example, through discovery via the World Wide Web. Moreover, beliefs about the new external state can exist, at least partially, only in the mind of a person who envisages a future external state: for example, a future external state in which a startup has been founded and has grown into a large organization with global reach. Thus, the formulation of an internal generative model can involve imagining a future external state. In particular, a person can imagine themselves in the future within the imagined future external state [50]. Through this sophisticated active inference, a startup founder can consider simultaneously “what would happen if I did that” and “what I would believe about what would happen if I did that” [51]. Additionally, through theory of mind, the startup founder can consider what other people think about the action taken and what other humans would think about the startup founder for taking the action [52]. Thus, a startup founder can formulate internal generative models that can encompass alternative potential startups situated in future external states. These internal generative models can encompass alternative courses of action, such as market offerings of goods/services, founder’s own feelings about those market offerings, founder’s estimate of potential customers’ feelings about the market offerings, and founder’s estimate of the feelings of society about the startup founder and the startup.

Compared to detailed internal generative models for routine daily life, nascent internal generative models for alternative potential future startups can be lacking in detail. They can be conceptual models that need more sampling from the external state to become schematic models that can provide the basis for beginning lean startup by formulating falsifiable hypotheses about the startup ideas. Further sampling from the external state is required to embed these hypotheses into a business model. Then, more sampling is required to enable development of a minimum viable product in order to test the business model. By the fourth stage of lean startup, alignment of internal generative models with external states begins to involve actions in the external state. These actions in the external state encompass opportunity creation, effectuation and bricolage. In particular, interaction with earlyvangelist customers to get iterative feedback in development of the minimum viable product. That is, a version of the startup’s proposed marketing offering with just enough features to be usable by early customers who can then provide feedback for future product development. Subsequently, in the fifth state of lean startup, running tests, can involve aligning what has become a detailed internal generative model with the external state through changing what is sampled from the external state and through changing actions in the external state. For example, changing what is sampled by eliciting feedback from a wider range of potential customers about the minimum viable product, and changing actions in the external state by modifying the minimum viable product in response to their feedback. Subsequently, in order for the startup to survive through enacting its business model, the founder will need to exploit information gained through the preceding exploratory active inference [53] carried out during the five phases of lean startup activities.

**Figure 2:**
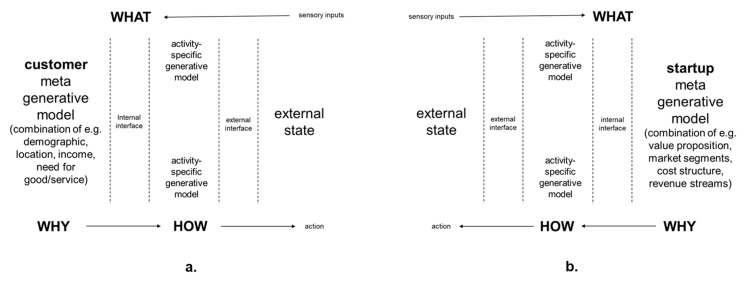
Meta generative model for intended customers as defined by startup (**a**) are aligned with (**b**) the startup’s own meta generative model developed during lean startup activities.

As shown in Figure 2 below ideally, after a startup has gone through the lean startup activities, it will have a meta generative model that is aligned with descriptions of its intended potential customers. Meta generative models encompass characteristics that are applicable to many different activities in many different situations. For example, the meta generative model of an individual person can include personality type, which underlies prior expectations during many different situations [54]. Meta generative models for startups’ potential customers can encompass descriptions of goals that they are attempting to accomplish, and their related needs for the startup’s good/service. Descriptions can include visual images that can distill multiple details including demographics, locations, occupations, hobbies, levels of digital literacy, disposal income, etc. As summarized in Figure 2, meta generative models are autopoietic models of self in the world and provide the basis for *why* actions are taken in the world. Activity-specific generative models provide the basis for *how* actions are taken in the world. For example, generative models for marketing management activities or generative models for operations management activities. Together with sensory inputs, these generative models, influence *what* sensory inputs are experienced from actions taken in the world. Such multi-level generative models are often referred to as being hierarchical in accordance with the notion of top-down expectations and bottom-up sensory inputs [54].

This model is hierarchical in the sense that the *why* states predict the *how* states, which themselves predict *what* sensory data. The inversion of this model (i.e., inference) then involves passing the *what* messages back up the hierarchy to infer the *how* states and, in turn, the *why* states. As is appropriate for a practitioner paper, the term meta generative model is used here colloquially. It is used to refer to an additional hierarchical level or factor in the generative model. The prefix ‘meta’- is used in the sense of ‘next to’. This can be regarded as a superordinate hierarchical level that is ‘next’ to the penultimate level in a hierarchical generative model. Alternatively, it can be regarded as an additional factor that is ‘next’ to the remaining factors that constitute the generative model. This meta level is meant to convey the inclusion of latent or hidden states that are conserved over time and contextualise (activity-dependent) state transitions in other parts of the generative model. Accordingly, meta generative model is used here to summarize the multitude of inter-related variables that can exert *why* influence over the *how* of activity-specific models. Overall, the more likely prior expectations in the meta generative model are considered to predict a sensory input (i.e., the higher the prior probability), the more attention will be paid to the prior expectation and the more influence the prior expectation will have on what is experienced. By contrast, the lower the prior probability, the more attention will be paid to the sensory input and any prediction error between prior expectation and what is actually experienced [54].

From the perspective of ACM, the startup meta generative model comprises decisions about the allocation of finite time and energy that begin the life history strategy of the startup. To the extent that the startup meta generative model is developed by one founder, it can be an expression of that person’s stress responsivity pattern (I, II, III, or IV), which reflects the life history strategy of that person so far. As the formulation of the startup meta generative model involves creative exploration of potential for survival and growth in future environments, it can involve filtering and/or amplification of opportunities and/or threats in those imagined future environments as they are sampled by the stress response system. If only one founder is involved, business development can involve only that person’s stress appraisals of alternative imagined future environments.

### 3.2. Transition: Interface between Internal State and External State

**Figure 3:**
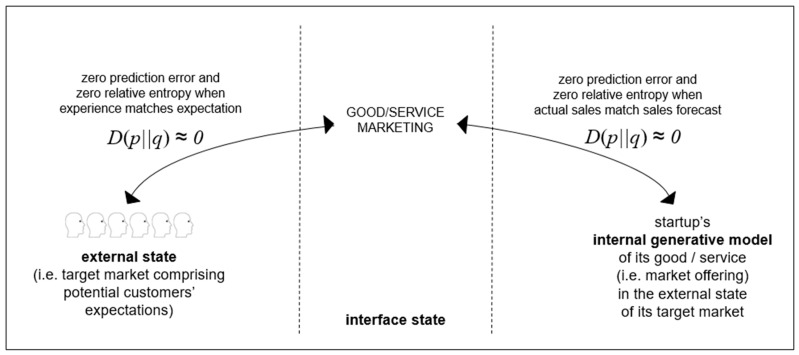
Startup interface state. Within the interface between the startup and its potential customers are the startup’s advertising of its good/service. If what is advertised is the same as what is produced by the startup, as defined in its good/service specification, there can be zero prediction error and zero relative entropy as customers’ experiences match their expectations from the startup’s advertising, hence actual sales match startup’s sales forecasts: i.e., internal generative model is aligned with the external state.

Interactions between the internal generative model and the external state takes place across the interface state (i.e., Markov blanket state [31]). For startups, transition towards scaling up takes place across the interface state through marketing that involves analyzing market characteristics, formulating market offerings, and adapting market offerings in relation to market responses. Ideally, marketing will lead to the situation summarized in Figure 3 below, where there is zero prediction error and zero relative entropy between sales forecast (i.e., what is predicted to happen) and actual sales (i.e., what happens). Probabilities can be allocated to predictions and probabilities can be allocated to events as they are happening. For example, the final value of some sales orders may only be definite months after the initial order has been received: meanwhile probabilities can be allocated to the total sales values. Relative entropy (*D*), sometimes referred to as Kullback–Leibler divergence, is a measure of differences between a reference probability distribution (e.g., a sales forecast) and a related probability distribution (e.g., total sales values). Zero prediction error and zero relative entropy can be achieved through the marketing of the startup’s good/service matching the specification for the good/service. This can lead to the situation where market sales match the startup’s sales forecast. However, poor marketing, such as poor pricing of market offerings, is often a cause of startup failure [2].

AIT can be related to the failure of startups offerings to survive in markets in terms of expectation disconfirmation [55]. In particular, the startup’s marketing can lead to potential customers having positive beliefs about the startup’s offering. Potential customers’ positive beliefs can lead to them having positive expectations about trying the startup’s offering. Their belief-based positive expectations can lead to them make specific positive predictions about what they will experience when trying out the startup’s offering. However, if their positive expectations are not confirmed when they take action to try the startup’s offering, they experience an unwanted surprise from the prediction error about would be experienced. The resulting actions of the customer, which are contrary to what the startup’s internal model predicted, in turn causes unwanted surprise for the startup. In particular, it is unwanted surprise for the startup because the startup believes in its market offering and has predicted positive cash flows from its market offering because it expects potential customers to try it out and want to experience it again in the future. Moreover, many potential customers experiencing unwanted surprise from the startup’s offering threatens the startups survival because it will not have incoming revenue from sales to cover outgoing costs from its operations. Rather, the startup will have unsustainable negative cash flow.

**Figure 4:**
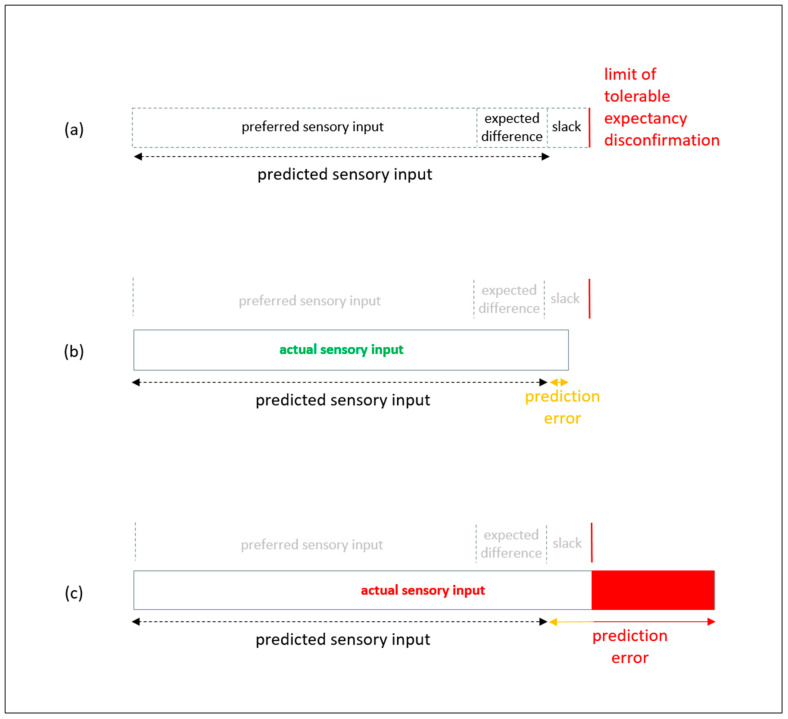
Limit of tolerable expectancy disconfirmation from startup’s market offering. (**a**) predicted sensory input (preferred sensory input plus expected difference between preferred sensory input and actual sensory input); (**b**) tolerable prediction error as sensory inputs are farther from preferred than expected but are within limit of tolerable sensory input from unwanted surprise; (**c**) not tolerable prediction error as sensory inputs are farther from preferred than expected and are beyond limits of tolerable sensory inputs from unwanted surprise.

As shown in Figure 4 below, the limit of tolerable expectancy disconfirmation can comprise two sources. First, expected difference between preferred sensory inputs from the startup’s market offer and actual sensory inputs from the startup’s market offer. Second, some additional tolerance for unwanted surprise, which can be described as float or slack on an action [56]: in this case, the action of trying out the startup’s market offering. If an action is carried out many times, limits of tolerable expectancy disconfirmation can be defined in statistical process control (SPC) charts [31]. However, if the action is a one-off, for example, during the trial of a startup’s good/service by a potential customer, then the limit of tolerable expectancy disconfirmation is a one-off. If the potential customer expects there to be no difference between preferred sensory inputs and actual sensory inputs, then the expected difference is zero. Moreover, the potential customer does not expect there to be any information gap between her internal generative model for using the startup’s market offering and actually using it in the external state of the world.

Expected difference and limit of tolerable expectancy disconfirmation can be related to AIT in terms of expected free energy (EFE) and variational free energy (VFE) upper bound. VFE can be considered as information gap between agents’ internal models and reality [30], which varies as internal states change and/or external states change. If the VFE upper bound, evaluated at some sensory input, is very large the information gap between internal model and reality can be too wide to be sustainable. Accordingly, within AIT, loosely speaking, agents select sequences of actions (i.e., policies) that will bring about future observations that minimize VFE. As future outcomes are yet to be observed, actions need to be selected that can minimize expected free energy (EFE): i.e., expected information gap between internal model and reality after action has been taken. Conceptually, the EFE can be related to the VFE by noting that VFE can be expressed as complexity minus accuracy (i.e., minimizing free energy leads to the most accurate but minimally complex explanation of the world). In the EFE the accuracy term of the VFE is replaced by a negative ambiguity, and the complexity term is replaced by risk. This means that the expected free energy favors minimally ambiguous and minimally risky futures. Action selections intended to minimize EFE seek to resolve uncertainty and to maximize reward. Actions will tend to be exploratory when beliefs about states are very uncertain. Conversely, action selections will tend to exploit information, which has been gained during exploration, in order to maximize reward when confidence in beliefs about states is high. It is important to note that VFE, and related constructs, such as EFE, do not have the same measurement units as sensory inputs. Rather VFE is a function of sensory input in the space of log probabilities.

As shown in Figure 4a, a potential customer for the startup’s offerings may expect some difference, and also have some additional tolerance for more than expected difference: i.e., some additional float/slack for unwanted surprise from an action. The term, tolerance, is not an integral feature of AIT. Rather, it is a term that is widely used in industry. Here, tolerance means the difference between the prediction and maximum permissible error. In SPC charts for repetitive processes, tolerance is between process mean and process control limits. Tolerance corresponds to the difference between prediction and what can be described, in AIT terms, as maximum negative evidence for the internal generative model [31]. This additional tolerance for unwanted surprise can arise from the startup’s competitors’ existing market offerings not providing the potential customer with preferred sensory inputs. Consider, for example, a potential customer in Africa who survives by making deliveries with rented old bicycles [57]. This is extremely strenuous work involving thousands of peddling and pushing actions that can consume thousands of calories of energy. The person survives through a precarious daily balancing of energy expenditure and energy consumption. Survival depends on earning more money from making deliveries than is needed to pay bicycle rental charges and is needed to buy food calories for peddling and pushing the rented bicycle.

The person may have mixed expectations about a startup’s market offer of rental cargo bicycles that are powered by electric batteries (e-bike) [58]. In particular, the person may predict rental costs to be higher but predict this to be more than off-set by reduction of current costs from buying food to get energy to peddle and push conventional bicycles. As summarized in Figure 4a, tolerance for unwanted surprise is minimal. This is because the person has minimal surplus resources available to cope with unwanted surprise [59].

In Figure 4b, sensory inputs from trying out the e-bike are not as expected but within tolerable limits: e.g., better than fatigue from making the same deliveries with the conventional old rental bicycles. This is because the same deliveries were made and the same money paid, but the amount of peddling and pushing was less. By contrast, in Figure 4c, sensory inputs from trying out the e-bike are worse than tolerable limits: e.g., worse than the fatigue from making the same deliveries with the conventional old rental bicycles. This is because use of the e-bike did not reduce the amount of peddling and pushing actions sufficiently to offset its extra rental costs. Hence, there was almost the same amount of peddling and pushing but less money available to buy food calories. Thus, tolerance for expectancy disconfirmation can involve comparisons between alternative internal generative models. In this case, internal generative model for continuing with renting old bikes versus internal generative model for beginning to rent e-bikes. However, when life takes place in a precarious balance between energy input and energy output, the limit of tolerable expectancy disconfirmation is existential: i.e., survival is not possible beyond the limit.

As the person survives through a precarious daily balancing of energy expenditure and energy consumption, any additional energy consumption involved in making the same deliveries can threaten the person’s survival through a vicious cycle in which not being able to complete deliveries due to exhaustion will result in being paid less and having less money to buy food. This example illustrates that survival can depend upon resolving information gaps between internal generative model and external state. In which case, the information gap between preferred sensory input and limit of tolerable expectancy disconfirmation can be described as survival information deficit. Uncertainty about the ways in which this survival information deficit could be addressed are a source of existential information entropy. For example, the delivery person may speculate about different ways that the e-bike could be otherwise be used to increase deliveries made and/or reduce energy expenditure from peddling and pushing. However, any speculative probabilities assigned to these speculative options are associated with mismatches between energy input and energy output that cannot be survived.

If the majority of potential customers experience prediction errors about the startup’s e-bike offering no worse that as summarized in Figure 4b, that offering can provide the basis for transition towards scaling. By contrast, it cannot provide the basis for transition towards scaling if the majority of potential customers experience prediction errors that are beyond their limits of tolerable expectancy disconfirmation as summarized in Figure 4c. In which case, the startup should update its meta internal generative model through double-loop learning [38] informed by its first-loop learning from negative customer feedback. For example, its value proposition should be revised.

At the same time, through ACM double-loop learning, the startup may re-calibrate its stress responsivity pattern. For example, a startup founder with stress responsivity pattern I, sensitive, may move towards stress responsivity pattern III, vigilant, due to the perceived threat to survival in the intended business environment. However, this could lead to exaggerated stress appraisals. Moreover, if permanent, this could be counterproductive because in the vigilant pattern (III), psychological resources are employed to monitor and cope with possible sources of threat and/or social competition, rather than to maximize exploration and learning as in the sensitive pattern (I).

## 4. Scaling and Exit: Survival of the Fittest

### 4.1. Scaling: Maintaining Non-Equilibrium Steady State (NESS)

The internal states of startups need to survive in the external states of the world around them by the startups maintaining a non-equilibrium steady state (NESS). Maintaining a steady state involves a startup’s internal state not dissipating into the external state. For example, a startup can maintain a steady state through its operations management ensuring that its internal resources are not scattered into the external state by being taken into the possession of external creditors via bankruptcy proceedings. Maintaining a non-equilibrium steady state involves the startup being able to continually adapt internally in relation to changes in the external state. Internal adaptations can be reactive in response to changes in the external state and/or proactive to bring about changes in the external state. Non-equilibrium steady states can range from near-equilibrium, which is maintained by homeostasis, to drifting towards far-from-equilibrium, which is addressed by allostasis [29]. Survival as the ecologically fittest depends upon making adaptations that are better than those of competitors in the same environment. For a startup, internal adaptations can include improving business processes, and external adaptations can include improvements to enabling infrastructures for the operation of its market offerings.

Homeostasis can be described as being a first-order feedback loop that regulates essential variables in near-equilibrium steady states. For example, a startup ensuring that outgoing costs are at least matched by incoming revenues through diligence in routine actions such as sending out invoices. In terms of organizational studies, refinements to processes such as, for example, sending out invoices, come from single-loop learning [38]. Allostasis can be described as a second-order feedback loop that reorganizes a system’s input–output relations when first-order feedback has failed, and the steady state is moving towards far-from-equilibrium. Allostatic reorganization being done to restore a near-equilibrium steady state by reestablishing stability of essential variables. In terms of organizational studies, allostasis is analogous to double-loop learning [38]. Allostasis involves the stress response system which coordinates allocation of time and energy to different activities that are intended to promote ecological fitness [60]. That is, promote the compatibility of a living thing with the environmental niche in which it intends to survive [61]. It is very important to avoid allostatic overload arising from second-order feedback loops being continually overtaxed. This is because allostatic overload can contribute to burnout [62], which is a common cause of startup failure [2,63]. Moreover, allostatic overload can contribute to the collapse of higher goals [29]. Apropos, there are many ways that a startup can reorganize its input-output relations, including changing its markets by pivoting to another business model [64].

However, the homeostasis and allostasis of startups can be affected by biases [65]. These can include initial overconfidence followed by escalation of commitment to failing courses of action. Such biases are examples of an internal generative model determining attention, expectation, and action irrespective of sensory inputs coming from the external state [54] such as negative sensory inputs from market analyses. Importantly, internal generative models can be affected negatively by stress [30], and stress is common among startup founders [63]. In accordance with ACM, and within AIT, updating the internal generative model can be based on the action of changing what sensations are sampled from the external state. For startups, this can include stopping paying attention to sources of negative market signals and carrying on with the same course of action based on the erroneous expectation, which has been generated by the biased internal model, that this will lead to survival. In terms of ACM, this could involve re-calibration to stress responsivity pattern IV, unemotional. If permanent, this would be counterproductive because pattern IV involves generalized unresponsivity that inhibits social learning and sensitivity to social feedback, which can also increase risk-taking by blocking information about dangers and threats in the environment. Negative market signals can include lack of interest among potential customers for the startup’s market offering.

In the e-bike example, there could be several reasons why the e-bike did not reduce the amount of peddling and pushing sufficiently. For example, it could be because the e-bike is not suitable for hilly terrain in a country such as Burundi [57]. Also, additional peddling and pushing could be required to get to battery recharging center where empty batteries are returned and charged batteries are collected. Accordingly, the startup could try to survive by preventing dissipation of its non-equilibrium steady state (NESS) through allostasis involving reorganizing its input–output relations. This could involve niche construction and participation in wider ecosystem engineering. In other words, allocating finite time and energy primarily to construction actions.

Niche construction involves adaption of the environment to better enable survival [66]. This can involve adaptive preferences [67] where people forego a first preference in order to survive. For example, people prefer to live above ground, but people will adapt this preference and modify the environment by building underground settlements if that can fulfil their primary goal of surviving [68]. On a wider scale, ecosystem engineering can involve more far-reaching changes to the environment that alter survival pressures positively for the ecosystem engineers but negatively for others [69]. Thus, there can be eco-evolutionary feedbacks in ecosystem dynamics [70]. This can lead those that are negatively affected to disperse. This happens when the expected ecological fitness benefits of moving outweigh the expected ecological fitness costs of moving [71,72].

In the e-bike example, niche construction by the startup could include moving its operations to a region with flat terrain and setting up one e-bike center from which its e-bikes are rented and repaired, and where the e-bike batteries are recharged and replaced. Then, in order to reduce the time and energy spent by customers in travelling to a from the one e-bike center, the startup could extend its niche construction by arranging for some of its services to be carried out at small roadside shops. For example, the startup could deliver charged batteries to the roadside shops and collect empty batteries left by their customers. Wider ecosystem engineering could involve the startup working with other organizations involved in introducing battery power for other machines to setup multi-machine battery charging points at multiple locations. Additionally, the startup could work with other organizations to support the introduction of renewable energy sources for battery charging points [73,74]. Such allocation of time and energy to construction and cooperation in competition against established vehicle rental companies could lead to maintaining NESS and scaling of the startup’s operations towards the exit stage of its life cycle.

### 4.2. Exit: Relative Ecological Fitness

Ecological fitness can be absolute and relative. Absolute fitness refers to ability to survive in an environment. Relative fitness refers to ability to survive better than others in an environment. Numerically, relative fitness ranges from anything above 0 to 1: with best relative fitness being 1 [61]. For the most favorable initial public offering of shares, private sale, merger, or acquisition, a startup needs to show that it is on its way to establishing best relative fitness.

For example, e-vehicle niche construction and ecosystem engineering could lead to the dispersal of established vehicle rental companies to other regions, such as hilly regions where the electric power of e-bikes may not be adequate. However, as there are already established vehicle rental companies in those regions, survival may not be possible through geographical relocation. Rather, the favorable landscape for established vehicle rental companies can first become fragmented by the niche construction of e-vehicle companies. Then, further niche construction for e-vehicles can lead to loss of favorable habitat for established vehicle rental companies as their landscape splits into patches, which little by little shrink and become more isolated from each other [75].

By contrast, as e-vehicle technology improves, wider ecosystem engineering can lead to the new e-vehicle niches becoming less isolated and more interconnected into a new e-vehicle landscape that encompass hilly as well as flat terrain. Hence, the NESSs of established vehicle rental companies becomes harder to maintain, and it becomes more likely that their internal resources will be scattered into the external state by being taken into the possession of external creditors via bankruptcy proceedings. However, while the landscape and NESSs of established vehicle rental companies may have survived many previous decades, the landscape and the NESSs of e-vehicle rental companies may be more short-lived as niche construction for bikes powered by hydrogen fuel cells begins [76].

**Figure 5:**
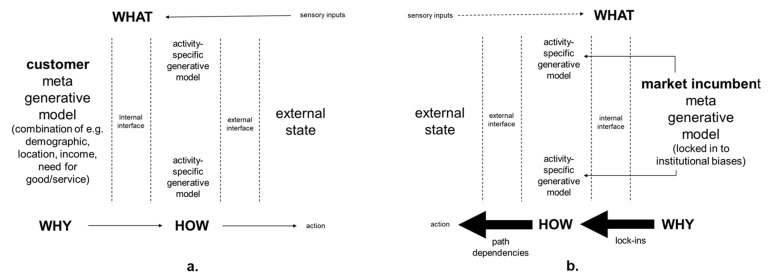
Evolving meta generative model of (**a**) customers is not matched by (**b**) market incumbent’s meta generative model that is locked-in to its biases.

As summarized in Figure 5 below, market incumbents, such as established vehicle rental companies, and possibly e-vehicle rental companies in the near future, may not change in response to new market expectations because people working in market incumbents can have biases that lead to them only sampling inputs from the external state sensory that support their biases [77,78].

Biases throughout the organizations being based on, for example, lock-ins to past investments that lead to persisting with path dependent actions even when there is sensory evidence that they are no longer effective [79,80]. People and organizations may not recognize their own biases, and their biases can be difficult to reduce even when they are recognized [81,82]. Consequently, maintaining the internal state in the external states of the world can be undermined by the market incumbent’s actions in the world being determined by an out-of-date meta generative model rather than sampling from the external state of the changing world. Importantly, this can prevent a favorable exit if electric power is already perceived as going to be superseded soon, for example, by hydrogen power.

Accordingly, to avoid loss of relative fitness that can undermine the exit stage, it is important to maintain stress responsivity pattern I, sensitive, or stress responsivity pattern II, buffered, in order to enable openness to the physical and social environment as it changes. Rather than the fight/flight responses predominating in the vigilant pattern (III) or the unresponsivity of the unemotional pattern (IV). Neither of which, if permanent, may enable balanced stress appraisals and learning needed to update internal generative models in changing environments.

## 5. Future-Proofing Principles for Startups

ACM and AIT are based on living things that have evolved through many millennia to survive within a few types of natural environments that change little from one generation to the next. By contrast, human niche construction and ecosystem engineering can bring many environmental changes within one generation. Some of these changes are unintended, negative, and difficult to control [83]. Moreover, climate-related environmental changes are becoming more widespread [5,6,7,8,9,10]. Accordingly, maintaining the NESS of human organizations in the 21st century and beyond can depend on changing the ratio between exploration for information and exploitation of that information. In particular, more time and energy may need to be spent on exploration followed by there being less time to exploit the information gained during exploration. Moreover, maintaining the NESS of human organizations in the 21st century and beyond can depend on being able to cope with shorter periods of homeostasis and more instances of allostasis.

Hence, a first organizing principle for future-proofing is for startups to carry out formal definition of their stress responsivity patterns. This can begin with startup founders defining their own ACM stress responsivity patterns. This can be carried out, for example, during SWOT analyses that define strengths and weaknesses in relation to opportunities and threats [84]. There is no one best stress responsivity pattern. Rather, a stress responsivity pattern can be a strength or a weakness depending upon the opportunities and threats in the environment. Accordingly, startups need to assess the compatibility of their stress responsivity pattern with the opportunities and threats in the environment. As shown in Figure 1, the stress response system filters and/or amplifies threats in relation to support that can offset them. However, either too little or too much support can undermine the potential for the stress response system to enable ecological fitness [22].

**Figure 6:**
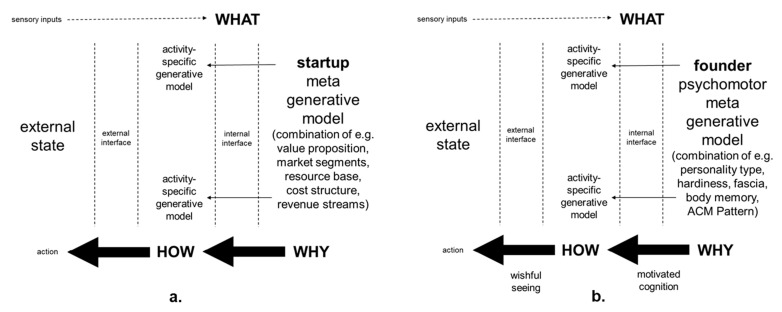
Startup meta generative model (**a**) based on motivated cognition and wishful seeing arising from (**b**) startup founder’s psychomotor meta generative model including ACM stress responsivity pattern (I, II; III or IV).

A second organizing principle is for startups to address the potential of their stress response systems to exert determining influence over sampling from the environment during business model development. This can be addressed by making AIT an explicit process in which the relationship between internal generative models and sampling from the environment is recognized. In particular, as summarized in Figure 6 below, the potential for founders’ internal meta generative models, including their stress responsivity patterns, to lead to motivated cognition and wishful seeing [77,78], should be recognized. This is important because it can lead to their startup’s business models being based on biased sampling that confirms their founders’ preconceptions and their habitual stress appraisals. Rather than being based on alignment with the expectations of potential customers as shown in Figure 2.

As startups move to their transition stage, they move from informal structure towards formal structure. Apropos, a third organizing principle is for startups to move from its stress response system being that of its founder to it being a set of well-resourced work procedures [27,36], which can emulate the roles of the sympathetic system, the parasympathetic nervous system, and HPA.

Beginning during the transition stage, a fourth organizing principle for startups is to check the compatibility of their stress responsivity patterns with changing external environments. In particular, check the extent to which double-loop learning has led to the stress responsivity pattern being well aligned with the current environment or has led to it being badly aligned by being fixated on past environments [85]. In terms of organizational studies, explicit consideration of the effects of implicit double-loop learning can be described as triple loop learning [86]. In doing so, startups can consider what is their own limit for tolerable expectancy disconfirmation. As summarized in Figure 4, like their customers, startups can expect some disconfirmation and can have some slack resources to deal with expected disconfirmation and unexpected disconfirmation [87,88]. However, startups cannot have infinite slack resources and need to recognize that unwanted surprise from the environment that exceeds limit of tolerable expectancy disconfirmation will undermine survival.

As startups begin their scaling stage toward exit stage, during which functional specialists take roles once covered by generalists, a fifth organizing principle is to check that the formal stress response system that is documented in work procedures is not superseded in practice by the human stress response systems of the functional specialists who are appointed during crises of leadership and bureaucracy [32].

During exponential growth of scaling towards the exit stage, it is essential to ensure that the resources required to do work in accordance with customer expectations does not exceed the resources available to the startup. This is important to prevent organizational stress that can lead to the collapse of higher goals such as ethical standards [89]. The amount of resources required to do work in accordance with customer expectations can be exceeded if an accumulation of individually small organizational errors leads to the startup being overwhelmed by what can be described as firefighting: in other words, becoming trapped in a quagmire of deadline pressure, overtime working and energy depletion [31,90]. This represents a failure of homeostasis’ first-order feedback loop that regulates essential variables in near-equilibrium steady states. It also represents a failure of stress response system’s co-ordination of allostasis’ second-order feedback loop that reorganizes input–output relations when first-order feedback has failed. This is because organizational firefighting moves organizations closer to, rather than away from, far-from-equilibrium. Accordingly, particularly during exponential growth of scaling towards the exit stage, startups need to have stress response systems that can address emergent weaknesses of internal operations. Especially, emergent weaknesses that can undermine the stability of essential variables, such as cash flow, which are needed to regulate traits and behaviours affecting ecological fitness [22]. Hence, a sixth organizing principle is that the stress responsivity pattern needs to be updated through explicit AIT processes to ensure it can address emergent weaknesses of internal operations during exponential growth.

**Figure 7:**
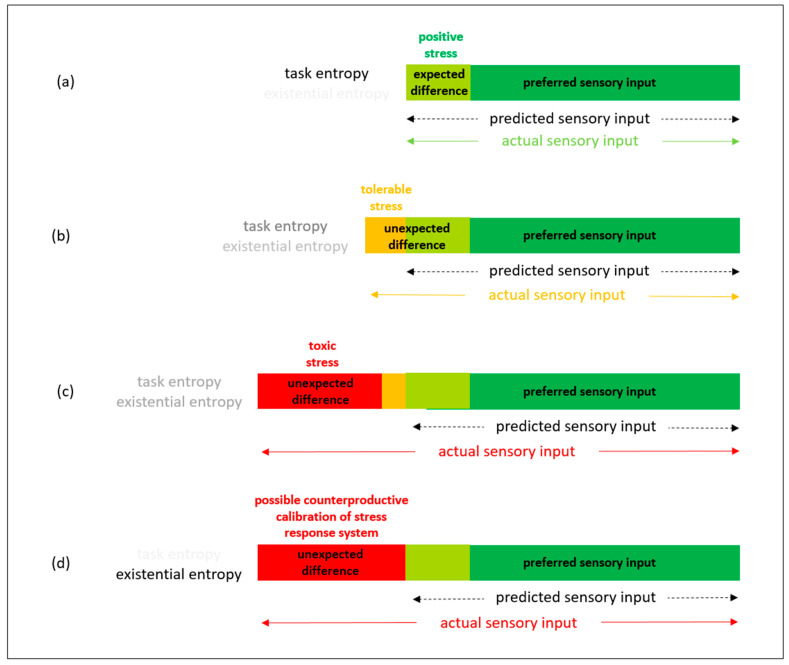
From task entropy to existential entropy. (**a**) Expectancy disconfirmation within range of expected difference: information entropy arises from work tasks. (**b**) Within limit of tolerable expectancy disconfirmation: information entropy arises from work tasks but with more uncertainty. (**c**) Beyond limit of tolerable expectancy disconfirmation: information entropy arises from survival uncertainty. (**d**) Beyond limit of tolerable expectancy disconfirmation: existential information entropy arising from increased survival uncertainty can lead to counterproductive calibration of stress response system.

A seventh organizing principle, which encompasses all stages of the startup lifecycle, is to avoid toxic stress that can lead to stress response system maladaptation. In particular, maladaptation through not recalibrating/updating stress responsivity pattern or maladaptation from recalibrating/updating to a stress responsivity pattern that is not congruent with the environment. In order to avoid toxic stress, it is necessary to maintain at least the minimum resources required to enable ecological fitness: i.e., to enable survival in operating markets. This involves the response to the loss of resources, for example from prediction errors, being to carry out resource development to replenish and/or enhance resource stocks. Resource development to prevent toxic stress need not involve large financial expenditure. For example, it can include taking rest, social support, and/or refreshing skills [91,92]. This proactive response to resource loss can be formalized as an explicit policy for active inference by the startup: initially by its founder and subsequently by its management. This can be done with the rationale that replenishing resources reduces potential underlying stress about survival. Thus, while there can be uncertainty, and associated information entropy, about the ways in which resources will be deployed in order to survive, this is not uncertainty about survival itself and the associated information entropy is not existential information entropy that can lead to maladaptive stress. As summarized in Figure 7 below, maintaining resources is necessary to reduce potential for expectancy disconfirmations to take stress beyond positive stress and tolerable stress towards toxic stress [93]. Figure 7a summarizes actual sensory inputs being within expected difference from preferred sensory inputs: i.e., sensory inputs are within the expected range. In such scenarios, information entropy arises from specific uncertainties about particular work tasks [94]: not from uncertainties about survival. There is no need for the startup to draw upon its spare resources (i.e., slack), and stress can be positive involving brief mild stress responses. Figure 7b summarizes actual sensory inputs going beyond expected range but within the scope of the startup’s spare resources. In such a scenario, stress can be tolerable if it is intense but temporary. Figure 7c summarizes actual sensory inputs going beyond expected range and beyond the scope of the startup’s spare resources that have already been reduced by previous prediction errors. In such a scenario, stress can be toxic if it is prolonged. Figure 7d summarizes actual sensory inputs going beyond expected range after all the startup’s spare resources have been consumed by dealing with its previous prediction errors. Loss of resources can increase stress because there are fewer resources available to enable ecological fitness [91,92]. The more prediction errors that are made and the more resources are lost, the more uncertainty there will be about the startup’s survival and higher potential for existential information entropy that can lead to maladaptive stress [29,30]. In such a scenario, stress can be more likely to lead to a counterproductive calibration of the stress response system.

**Figure 8:**
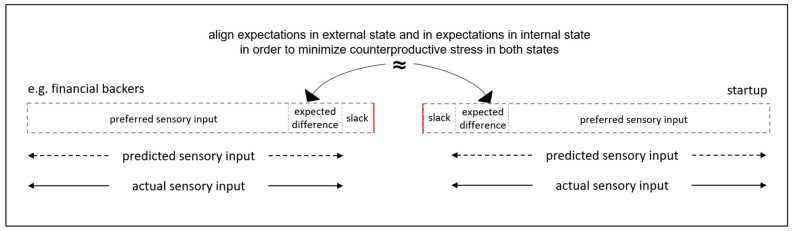
Manage expectations between internal state of the startup and the external state including, for example, financial backers, to minimize stress that can arise from survival uncertainty.

As summarized in Figure 8 below, an eighth organizing principle, which also encompasses all startup lifecycle stages, is to manage expectations. In particular, to align external expectations with internal expectations in order to avoid, what can be described as expectancy discrepancy/violation/disconfirmation [55,95,96]. For example, align customers’ expectations for good/service with startup’s specifications for good/service. Also, to align financial backers’ expectations for sales growth with startup’s plans for sales growth. The alignment of expectations can minimize the startup’s uncertainty about how it will survive. This is because there is minimal information gap between the startup’s internal generative models and the external state. When there is minimal information gap, any prediction errors will be within the range of expected difference from preferred sensory inputs, and stress will be minimal [29,30]. In terms of organization studies, the startup will not be stressed commercially or financially because sales forecasts will be reached, and cash flow forecasts will be within expected range of deviations. In terms of AIT, this corresponds to actual posteriors (e.g., sales income) matching expected posteriors (e.g., sales forecast) with free energy related to actions in the sales policy matching expected free energy, and being less that the variational free energy upper bound [31]. When there is minimal information gap, which is within expected range of deviations in sales forecasts and cash flow forecasts, any stress that is experienced in dealing with sensory inputs from customers and funders can be brief mild stress that can be described as positive stress [93]. Expectations should be updated and realigned in response to the loss of resources and/or the development of new resources in order to minimize potential for future expectancy discrepancies/violations/disconfirmations [55,95,96].

## 6. Conclusions

### 6.1. Principal Contributions

There are three contributions from this paper. The first contribution is to address the need for future-proofing methods for startups. This has been done through the definition of the eight organizing principles based on ACM and AIT that are summarized in Table 1 below. Together, these eight stress management principles go beyond previous literature concerned with entrepreneurial stress and organizational stress. In particular, rather than focusing on particular stressors, the principles are concerned with the recalibration/updating of startups’ stress responsivity patterns to changes in the internal state of the startup and/or to changes in the external state: instead of stress responsivity patterns being based on previous internal states and/or external states. In doing so, the eight principles address startups’ two fundamental growth challenges amidst dynamic environmental change [5,6,7,8,9,10]: arrival as the fittest and survival as the fittest.

A second contribution is relating ACM and AIT to four life cycle stages of startups. In doing so, overlaps between ACM and AIT are apparent. In particular, both encompass internal models that can have a determining influence over sampling from the external state and actions taken in the external state. Furthermore, both encompass internal models being changed by what can be described as double-loop learning [38]. Within ACM, this is the recalibration of stress responsivity patterns (I, II, III, IV). Within AIT, this is the updating of internal generative models. Within both ACM and AIT, double-loop learning does not necessarily lead to better alignment of the internal state with the external state. Rather, double-loop learning can be maladaptive [22,29,30]. From an organizational practice perspective, both ACM and AIT are pertinent to phenomena such as motivation cognition, wishful seeing (Figure 6), lock-ins and path dependencies (Figure 5) [77,78,79,80], within which preconceptions in internal models can override sensory inputs from the external state. When individuals and/or organizations get so stuck in their preconceptions, interventions can be required to get them unstuck [30,81,82]. This can involve reflection that can lead to triple-loop learning [86].

The third contribution is to provide practical examples that show the broader relevance ACM and AIT to organizational practice. This has been done with examples across the life cycle stages of startups, which are also relevant other types of organizations. These include examples related to business model development (Figure 2), marketing (Figure 3), customer experience (Figure 4), and organizational firefighting (Figure 7). In addition, the examples illustrate the congruence between life science theories and practice in human organizations. For example, the congruence between survival through minimizing long-term average surprise and organizations minimizing expectancy discrepancy, expectancy violation, and expectancy disconfirmation [55,95,96]. Here, it is important to note that there is ongoing debate about the exact meaning of terms, such as EFE and VFE, and interrelationships between them [97] Accordingly, there is not fixed exact correspondence between technical terminology and practice examples [98]. Overall, the paper makes a contribution to relating physics of life constructs concerned with energy, action and ecological fitness to practice in human organizations.

### 6.2. Directions for Further Research

Further research can encompass action research focused on implementation trials for the eight principles summarized in Table 1. Particularly relevant are startups that involve individuals and organizations that have developed in very different environments to have different stress responsivity patterns, and so can make different stress appraisals of the same events. For example, very different environments in northern Europe, where resources are plentiful, and southern Africa where resources are scarce. Particularly relevant are startups that involve the conversion and/or transportation of physical matter amidst the physical challenges brought by climate change. This is because of the potential for increased incidence of uncontrollable events that unpredictably increase expenditure of energy needed to survive. For example, increased energy required to survive as a startup involved in the delivery of physical goods by e-vehicles. As summarized in Figure 7, when the expenditure of energy and other resources repeatedly exceeds expectations, it is more likely that toxic stress will lead to counterproductive calibration of stress responsivity patterns. This could be particularly problematic when startups involve individuals and organizations that have different stress responsivity patterns that lead to different sampling and stress appraisals from the same situations.

As well as enabling contributions to the future-proofing of startups, such action research could lead to findings relevant to research into stress appraisals in new environments [99], interactions between resource depletion and stress appraisals [100], and dynamics between uncertainty and anxiety [101]. More broadly, such action research could lead to findings that are relevant to research into joint agent-environment systems where environments change alongside agents—often due to the action of agents themselves [102]. In particular, there could be relevance to research that encompasses the influence of agents’ prior beliefs over their inference. For example, the potential for prior beliefs that poorly represent the environment to lead to false inferences [103], which could undermine survival. In turn, advances in these life science fields have potential to further inform the future-proofing of startups. For example, research into dynamics between uncertainty and anxiety suggests that adoption of belief structures and clear goals can constrain experience of uncertainty. Also, it suggests that the formulation of clear explanatory narratives can support transforming uncertainty into understanding [101]. Thus, action research into future-proofing startups could encompass the formulation of narratives that explain how belief structures and concrete goals relate to markets in which startups intend to survive. Such an exercise can be framed as an explicit updating of startups’ internal generative models with belief structures encompassing the definition of stress responsivity patterns.

## Figures and Tables

**Table 1:** Future-proofing principles based on ACM and AIT.

Startup Stage	Principle
Business model development	Define stress response system in terms of ACM stress responsivity patterns and assess strengths and weakness in relation to opportunities and threats
	2. Address potential of stress responsivity pattern to exert a determining influence over sampling from the environment by making AIT an explicit process
Transition	3. Move from stress response system being that of its founder to it being a set of documented work procedures with sufficient allocation of resources
	4. Check the extent to which double-loop learning has led to the stress responsivity pattern being well aligned or badly aligned with the current environment
Scaling towards exit	5. Ensure that the documented stress response system is not superseded in practice by the human stress response systems of human functional specialists 6. Through explicit AIT processes, ensure stress responsivity patterns can address emergent weaknesses of internal operations during exponential growth
All stages	7. Avoid stress response system maladaptation from toxic stress by always counteracting loss of resources through development of new resources 8. Align expectations in external state and in expectations in internal state in order to minimize counterproductive stress in both states

## Data Availability

Not applicable.
